# What is the influence of exacerbations on pulmonary function in pediatric and adolescent patients with severe asthma despite controller therapies?

**DOI:** 10.1002/clt2.70046

**Published:** 2025-04-04

**Authors:** A. Z. P. Brandão, L. M. L. B. F. Lasmar, L. M. A. S. Pertence, M. I. R. Vieira, G. B. Lasmar, V. O. Ganem, E. V. Mancuzo, M. V. N. P. de Queiroz

**Affiliations:** ^1^ Department of Pediatric Pulmonology Hospital das Clínicas Federal University of Minas Gerais Belo Horizonte Brazil; ^2^ Multidisciplinary Center for Difficult‐to‐Control Asthma Hospital das Clínicas Federal University of Minas Gerais Belo Horizonte Brazil; ^3^ Pulmonary Function Laboratory Department of Internal Medicine Hospital das Clínicas Federal University of Minas Gerais Belo Horizonte Brazil

**Keywords:** adolescent, asthma, child, exacerbation, respiratory function tests, spirometry

## Abstract

**Background:**

Although exacerbations are common in severe asthma, there have been few longitudinal studies evaluating their effect on lung function parameters. This study aimed to evaluate the impact of exacerbations on lung function in children and adolescents with severe asthma in Brazil.

**Methods:**

This was a prospective study in which lung function parameters—forced vital capacity (forced vital capacity [FVC]), forced expiratory volume in 1 s (forced expiratory volume in 1 s [FEV1]), the FEV_1_/FVC ratio, and the forced expiratory flow between 25% and 75% of FVC (FEF_25–75%_), each expressed as a percentage of the predicted value—were measured at 3‐month intervals for three years in 64 patients (6–18 years of age) with severe asthma. Multivariate regression models of longitudinal data were employed to assess the associations between exacerbations and other predictors show with lung function parameters.

**Results:**

The mean duration of prior use of an inhaled corticosteroid together with a long‐acting bronchodilator or other controller was 6.7 (SD 3.2) years. During the study period, 31 patients (48.5%) had exacerbations. We analyzed 479 pulmonary function tests and found no significant association between exacerbation and any of the lung function parameters: FEV_1_ (*p* = 0.90); FEF_25–75%_ (*p* = 0.73); FEV_1_/FVC (*p* = 0.29); and FVC (*p* = 0.51). Passive smoking and being female were associated with mean FEV_1_ values that were 9.89% and 7.32% lower, respectively.

**Conclusions:**

In children and adolescents with severe asthma who are using preventive treatment, exacerbations do not seem to be associated with impaired lung function.

## 1 INTRODUCTION

Asthma exacerbations have an impact on patient quality of life and on the use of health care services. However, their consequences might not be limited to those of the acute event. Even after the exacerbation has resolved, long‐term consequences, including a loss of lung function, are possible.^1^, ^2^, ^3^, ^4^

A decline in function resulting from severe asthma exacerbations is well documented in studies involving the adult population, in which the occurrence of such exacerbations has been associated with the progression to irreversible obstruction over time.^5^, ^6^ However, there have been only a few studies involving the pediatric population, and those studies have produced conflicting results.^7^, ^8^, ^9^, ^10^ In a study of pediatric patients with moderate or severe asthma who were using preventive treatment, no association was found between exacerbation and a loss of lung function.^7^ Similarly, in a study of pediatric patients with the mild form of asthma, even severe exacerbations were not found to lead to a decline in lung function among those using an inhaled corticosteroid (ICS).^8^ Conversely, exacerbation was associated with a loss of lung function in a study of children and adolescents with difficult‐to‐treat or severe asthma.^9^ Although a loss of lung function was also observed in a cohort of pediatric patients with severe asthma, the loss was associated with being male and with higher exacerbation frequency.^10^

Lung function in adulthood is partly determined by factors and events from childhood, including frequent asthma exacerbations.^11^ Reduced lung function during childhood increases the risk of developing chronic obstructive pulmonary disease (COPD), especially in individuals with severe asthma.^12^

The cohort of children and adolescents with severe asthma evaluated in the present study had been followed longitudinally, with a protocol based on the American Thoracic Society/European Respiratory Society criteria, for 15 years^13^ In a previous study conducted by our group,^14^ we found that the lung function remained constant over time in patients with severe asthma, even in those who were using high doses of an ICS, together with other controllers.

Even an optimized, personalized therapeutic approach has not been shown to prevent frequent exacerbations,^3^ and there is a risk of reduced lung function in children and adolescents with severe asthma.^9^, ^10^ There is also a scarcity of data in the literature on this topic. Therefore, the objective of this study was to evaluate the association between exacerbations and lung function in children and adolescents with severe asthma of the pediatric severe asthma cohort in Brazil.

## 2 PATIENTS AND METHODS

This was a prospective study, conducted over a period of three years, in which we analyzed 479 pulmonary function tests (PFTs) of 64 patients in the cohort of patients with severe asthma who were enrolled in the secondary public network known as the Wheezy Child Program.^15^ Since 2010, patients have been referred, exclusively by pediatric pulmonologists, from the Wheezy Child Program to the *Centro Multidisciplinar de Asma de Difícil Controle* (CEMAD, Multidisciplinary Center for Difficult‐to‐Control Asthma) at the Hospital das Clínicas of the Federal University of Minas Gerais, in the city of Belo Horizonte, Brazil. The methods by which this cohort was recruited and followed were described in a previous study.^14^

The Global Initiative for Asthma defined severe asthma as asthma that is uncontrolled despite adherence with maximal optimized high‐dose inhaled corticosteroid plus long‐acting *β*_2_ agonist (ICS‐LABA) treatment and adequate management of contributing factors or that worsens when high‐dose treatment is decreased.^16^ According to these criteria, we included children and adolescents aged ≥6 and ≤18 years, with a diagnosis of severe asthma objectively confirmed by the presence of reversible expiratory airflow limitation with the use of salbutamol, adherence rate ≥80% to treatment, adequate inhalation technique and treatment of comorbidities, and at least 6 months of follow‐up at Cemad (Multidisciplinary Center for patients with difficult‐to‐control asthma).

Patients under the age of six or over the age of 18 years with other chronic lung diseases, cognitive impairment, neurological diseases, or immunodeficiencies were excluded. According to age, ICS doses are considered high if >400 μg/day in 6–11 years and in those over 12 years if >800 μg/day of budesonide/equivalent.^16^

### 2.1 Procedures

In all consultations, patients were evaluated in accordance with the standardized care protocol of the facility.^14^ That included spirometry in order to determine the forced vital capacity (FVC), forced expiratory volume in 1 s (forced expiratory volume in 1 s [FEV1]), FEV_1_/FVC ratio, and forced expiratory flow between 25% and 75% of FVC (FEF_25–75%_), each expressed as a percentage of the predicted value.

### 2.2 PFTs

All patients underwent PFTs, performed with a KoKo spirometer (KoKo PFT, Longmont, CO, USA) and in accordance with the recommendations of the American Thoracic Society.^17^ The PFTs were performed during scheduled appointments, always at the same location and always in the morning. For the functional diagnosis of asthma, the FEV_1_, FVC, FEV_1_/FVC ratio, and FEF_25–75%_ were measured before and after administration of a bronchodilator (400 μg of albuterol by metered‐dose inhaler). A significant postbronchodilator variation was defined as an increase in the FEV_1_ of 200 ml or 12%. The PFT parameters are expressed as a percentage of the predicted values for age, sex, and height.^16^, ^17^, ^18^

### 2.3 Environmental control

The home environment was considered uncontrolled if there was a report of passive smoking in the home. In such cases, environmental improvement measures were recommended and the level of environmental control was reassessed in subsequent consultations.^14^, ^17^

### 2.4 Treatment adherence

In each consultation, potential difficulties on the part of the patient or guardian in relation to adherence were identified and the necessary guidance was given, adherence being reassessed in the subsequent consultation. In addition to the self‐reported adherence rate, the rate of adherence to the use of ICS was also determined by calculating the proportion of doses used in relation to the expected number of doses for each time period, on the basis of the dose counters of the devices or by counting the empty capsules (for the dry‐powder inhalers), in comparison with the dates on which the medicines were dispensed.^19^

### 2.5 Optimization of the therapy

At each appointment, medications and doses were adjusted according to the level of asthma control. Blood pressure, growth curves, body mass index, and clinical variables were monitored to identify any adverse effects of the therapy.^17^ The medications used were dispensed via the *Sistema Único de Saúde* (SUS, Brazilian Unified Health Care System). The patients were receiving one of two types of treatment: with a dry‐powder inhaler delivering a combination of budesonide and formoterol—Symbicort (AstraZeneca, Lund, Sweden) or Alenia (Aché Laboratórios Farmacêuticos S.A., Guarulhos, Brazil); or with a dry‐powder or metered‐dose inhaler containing fluticasone, combined with salmeterol (Seretide; GlaxoSmithKline, Stevenage, England), oral prednisolone (generic), omalizumab (Xolair; Novartis Biociências S.A., São Paulo, Brazil), montelukast (Montelair; Aché Laboratórios Farmacêuticos S.A.), or tiotropium bromide (Spiriva Respimat; Boehringer Ingelheim do Brasil Química e Farmacêutica Ltda, São Paulo, Brazil).

This study was approved by the Research Ethics Committee of the Hospital das Clínicas of the Federal University of Minas Gerais (Reference no. 4.048.940). Written informed consent was obtained from all participating patients and their parents or legal guardians.

### 2.6 Statistical analysis

Descriptive analyses were performed by calculating frequencies, means, medians, and standard deviations. Longitudinal profile graphs were constructed from all PFTs, stratified by the presence or absence of exacerbation. To assess factors associated with lung function over time, we used regression models in which the dependent variables were the PFT parameters (FVC, FEV_1_, the FEV_1_/FVC ratio, and FEF_25–75%_) and the independent variables were as follows: exacerbation; rate of adherence to treatment with ICS and other controllers; current age; sex; age at symptom onset; disease duration; age at initiation/duration of ICS use; need for intensive care unit (ICU) admission due to asthma; self‐reported passive smoking in the home; and parental history of asthma.

In the first model, we used longitudinal regression for panel data. The independent variables exacerbation and adherence to treatment with ICS and other controllers were evaluated at the same time as the PFT outcome variables. The aim was to determine whether or not a measurement obtained in a given period, such as FEV_1_ in month 3, is influenced by the independent variables exacerbation and adherence to treatment with ICS and other controllers in the same time frame. Therefore, the outcome variables and independent variables were evaluated at the same time points over the entire 3‐year study period.

In the second model, the analysis performed was repeated‐measures multilevel regression for panel data; that is, the independent variables (sex, current age, age at symptom onset, disease duration, age at initiation/duration of ICS use, need for ICU admission due to asthma, self‐reported passive smoking in the home, and parental history of asthma) are fixed and therefore, unlike the outcome variable of interest, do not vary over time. The objective of this model was to determine whether or not the initial condition of the patient influences the PFT parameters over the study period (3 years), providing a robust analysis of intra‐individual variations. In both models, values of *p* < 0.05 were considered statistically significant.

## 3 RESULTS

The initial sample comprised 139 patients and 512 corresponding PFTs. A total of 75 patients were excluded, for the following reasons: being over 18 years of age (*n* = 19); being under six years of age (*n* = 13); not appearing for follow‐up appointments (*n* = 17); being discharged to another facility for no longer meeting the criteria for severe asthma (*n* = 19); and having PFT results that were not considered reproducible or acceptable (*n* = 7). Therefore, the final sample comprised 64 patients and 479 PFTs, evaluated over a period of three years. The mean number of PFTs per patient was 7.5 (range, 5.0–8.0). Table 1 shows the general descriptive characteristics of the patients at enrollment in the study. As can be seen in the table, most of the patients were female, the mean age at the initiation of ICS was quite low, and most patients were using two or more controllers at study enrollment. The ICS‐LABA doses were high overall at all visits (954.7 ± 4.0 mcg). Only 5 (7.8%) patients used omalizumab.

**TABLE 1:** Characteristics of the patients with severe asthma at study enrollment.

Characteristic					(*N* = 64)					
Female, *n* (%)					34 (53.1)					
Age (years), mean ± SD					10.0 ± 2.9					
Age at symptom onset (years), mean ± SD					1.3 ± 0.2					
Duration of disease (years), mean ± SD					8.8 ± 3.1					
Age at ICS initiation (years), mean ± SD					3.3 ± 2.6					
Duration of ICS use (years), mean ± SD					6.7 ± 3.2					
ICU admission due to asthma exacerbation, *n* (%)					13 (20.3)					
Family history of asthma, *n* (%)					51 (79.7)					
Passive smoking reported, *n* (%)					17 (26.6)					
Treatment adherence (%), mean ± SD					92.0 ± 12.1					
Medication(s) used, *n* (%)										
Inhaled corticosteroid + LABA					64 (100)					
Inhaled corticosteroid + LABA + others^a^					26 (40.6)					
Inhaled corticosteroid daily dose, mean ± SD					946.1 ± 32.3^b^					
Baseline PFT parameter (% of predicted)										
	Median min/max				Mean ± SD					
FVC	89.9 (57.1–127.7)				90.4 ± 16.9					
FEV1	82.1 (34.8–123.3)				79.7 ± 19.9					
FEV1/FVC ratio	82.8 (48.9–119.0)				81.9 ± 12.2					
FEF25%–75%	64.0 (16.0–150.0)				68.1 ± 28.9					

Abbreviations: FEF_25–75%_, forced expiratory flow between 25% and 75% of FVC; FEV_1_, forced expiratory volume in 1 s; FVC, forced vital capacity; ICS, inhaled corticosteroid; ICU, intensive care unit; LABA, long‐acting *β*_2_ agonist; LTRA, leukotriene receptor antagonist; PFT, pulmonary function test.

^a^ leukotriene receptor antagonist, oral corticosteroid, Tiotropium bromide. Omalizumab (5 (7.8%).

^b^ The mean (SD) dose used in the 1° appointment was 954.7 ± 49.0 mcg; in the 2°: 959. ± 43.4 mcg; 3°: 954.7 ± 44.7 mcg; 4°: 940.6 ± 36.7 mcg, 5°: 945 ± 39.3 mcg; 6°: 964.1 ± 45 mcg and 7°: 900. 8 ± 39.8 mcg.

Table 2 shows the characteristics of the exacerbations. Although 31 (48.4%) of the patients experienced exacerbations during the study period, 20 (64.5%) of those patients experienced only one. None of the patients required hospitalization during the study period.

**TABLE 2:** Frequency of exacerbations over a 3‐year period among children and adolescents with severe asthma (*N* = 64).

Variable	*n* (%)
Presence of exacerbations	31 (48.4)
Number of exacerbations	
0	33 (51.6)
1	20 (31.3)
2	5 (7.8)
3	2 (3.1)
4	2 (3.1)
5	2 (3.1)

Table 3 shows the results obtained with the longitudinal regression model for panel data. Of the outcome variables analyzed (FEV_1_, FEF_25–75%_, the FEV_1_/FVC ratio, and FVC), none showed any significant association with the explanatory variables exacerbation and adherence to treatment with ICS and other controllers. This suggests that, within the 3‐year study period, these variables had no impact on lung function.

**TABLE 3:** Longitudinal regression model of panel data in the assessment of the lung function of 64 pediatric patients with severe asthma (479 measurements) over a 3‐year period.

Outcome variable	Independent variable	Regression coefficient (95% CI)	*p* ^a^
FEV_1_	Intercept (*β*_0_)	87.15 (78.20; 96.10)	<0.001
	Exacerbation^b^	0.06 (−1.93; 2.06)	0.949
	Adherence (%)	−0.05 (−0.14; 0.04)	0.282
FEF_25–75_	Intercept (*β*_0_)	84.78 (69.01; 100.5)	<0.001
	Exacerbation^b^	−0.61 (−4.12; 2.90)	0.735
	Adherence (%)	−0.12 (−0.28; 0.03)	0.122
FEV_1_/FVC	Intercept (*β*_0_)	84.22 (78.45; 89.98)	<0.001
	Exacerbation^b^	−0.68 (−1.96; 0.60)	0.298
	Adherence (%)	−0.01 (−0.07; 0.04)	0.608
FVC	Intercept (*β*_0_)	98.00 (88.80; 107.20)	<0.001
	Exacerbation^b^	0.71 (−1.38; 2.79)	0.506
	Adherence (%)	−0.06 (−0.15; 0.33)	0.212

^a^ Breusch–Pagan Lagrange Multiplier test and Hausman test.

^b^ Yes versus no.

Table 4 shows the results of the repeated‐measures multilevel regression for panel data. Passive smoking was associated with a 9.89% mean decrease in FEV_1_. Patients who reported exposure to secondhand smoke had significantly worse lung function. The mean FEV_1_ was 7.32% lower in the females than in the males, a statistically significant difference.

**TABLE 4:** Multilevel regression model of 479 spirometry exams in 64 pediatric patients with severe asthma.

Outcome variable	Explanatory variable	Regression coefficient	SE	Z*	p**	95% CI
FVC	Intercept (*β*_0_)	97.39	2.17	44.92	<0.001	(93.14; 101.64)
	Female gender	−8.16	2.98	−2.74	0.006	(−14.01; −2.32)
FEV_1_	Intercept (*β*_0_)	87.77	2.71	32.37	<0.001	(82.45; 93.08)
	Time^a^ (months)	0.14	0.05	2.54	0.011	(0.03; 0.24)
	Female gender	−7.32	3.24	−2.26	0.024	(−13.67; −0.97)
	Passive smoking^b^	−9.89	3.66	−2.70	0.007	(−17.06; −2.72)
FEV_1_/FVC ratio	Intercept (*β*_0_)	84.19	1.23	68.32	<0.001	(81.77; 86.60)
	Passive smoking^b^	−6.16	2.39	−2.58	0.010	(−10.83; −1.48)
FEF_25–75%_	Intercept (*β*_0_)	74.16	3.58	20.73	<0.001	(67.15; 81.7)
	Time^a^ (months)	0.30	0.09	3.20	0.001	(0.12; 0.48)
	Passive smoking^b^	−16.43	6.59	−2.49	0.013	(−29.35; −3.52)

Abbreviations: FEF_25–75%_, forced expiratory flow between 25% and 75% of FVC; FEV_1_, forced expiratory volume in 1 s; FVC, forced vital capacity.

^a^ Study period.

^b^ Reported at enrollment.

*Statistic of regression model analysis. **Probability of significance of the Z statistic.

The results obtained with the final model suggest that disease duration, sex, and passive smoking reported at enrollment are major predictors of FEV_1_ over time in children and adolescents with severe asthma. Specifically, we observed that FEV_1_ tends to increase over time but is significantly lower in females and in patients who report passive smoking at home.

**FIGURE 1:**
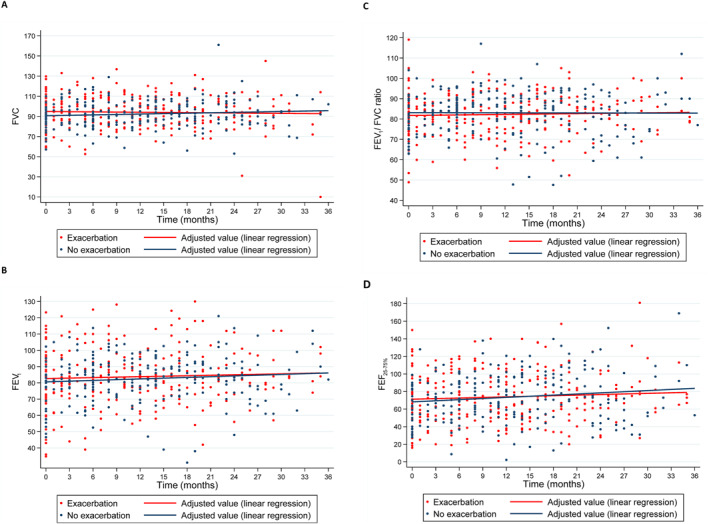
Evolution of the spirometry parameters forced vital capacity (FVC, in A), forced expiratory volume in 1 s (forced expiratory volume in 1 s (FEV1), in B), the FEV_1_/FVC ratio (in C), and forced expiratory flow between 25% and 75% of FVC (FEF_25–75%_, in D), in percentages of the predicted values, over a 3‐year period in pediatric patients with severe asthma, by the presence or absence of exacerbation.

Figure 1 shows the evolution of the PFT parameters over the 3‐year observation period, stratified by the presence or absence of exacerbation. Throughout the study period, those parameters did not differ significantly between the patients with and without exacerbations.

## 4 DISCUSSION

In this study, we demonstrated that, despite the use of high doses of ICS together with other controllers, exacerbations persisted in children and adolescents with severe asthma. However, we did not find the occurrence of exacerbations to be associated with a loss of lung function. Although the PFT parameters remained stable, lung function was lower in the patients who were female and in those who reported passive smoking in the home.

Therapy with increasing doses of ICS in combination with other controllers significantly reduces, but does not completely eliminate, exacerbations. Even with the use of the available biologics, the reduction in the annual rate of serious events is only 40%–50%. Therefore, a situation persists in which exacerbations are the “Achilles heel” of asthma and the best predictor of future serious asthma exacerbations is having had a recent serious exacerbation.^2^

In our sample of children and adolescents with severe asthma, exacerbations occurred in approximately half of the sample, although they were less frequent than before study enrollment (most patients had only one exacerbation during the study period), without the need for hospitalization and any impact on lung function. Notably, the other half of our sample remained controlled, but with high doses/age of medication associated with LABA, according to the definition of Severe Asthma.

Studies that have evaluated the association between asthma exacerbations and lung function parameters have varied in design, as well as in the duration of monitoring, severity of asthma, and exacerbation severity within their patient samples.^7^, ^8^, ^9^, ^10^ One study of patients with moderate or severe asthma evaluated FEV_1_ before and after each exacerbation over a 12‐month period.^7^ In that study, exacerbations occurred in one third of the patients, even though preventive treatment was used. The authors concluded that an asthma exacerbation does not lead to a lasting reduction in lung function. In the present study, we also demonstrated that exacerbations did not reduce FEV_1_, which remained stable over the 3‐year study period.

In the 3‐year cohort study known as the Severe Asthma Research Program (SARP) III,^20^ most of the patients had experienced at least one exacerbation in the 12 months prior to enrollment in the study. The authors detected an association between frequent exacerbations and significant decreases in FEV_1_, although only in the male patients. They concluded that frequent exacerbations during childhood have lasting effects on lung function. The negative effect is cumulative, and secondary prevention of exacerbations can improve lung function outcomes during childhood; therefore, asthma severity should be considered a useful indicator of lung function decline.^10^

The patients evaluated in the present study were from the CEMAD cohort and their characteristics were similar to those of the SARP III cohort in terms of age, disease duration, ICS dose, and number of controllers used. However, the SARP III cohort comprised more male patients, which may explain the sex‐related differences found. In addition, the mean FEV_1_ at enrollment was slightly higher in the SARP III cohort (87.4% vs. 79.7%) and exacerbations were less common in our cohort.

In a study involving pediatric patients with asthma exacerbations that required ICU admission, PFTs performed at 3–12 months and 12–24 months after discharge showed a reduction in FEV_1_. The authors concluded that children hospitalized due to asthma exacerbations that are more severe are at risk for a decline in lung function in the medium to long term after discharge.^21^ However, another study demonstrated that severe exacerbations, even in the context of mild asthma, were associated with a decline in FEV_1_ in those receiving a placebo, among children and adults but not among adolescents.^8^ Conversely, in the patients treated with budesonide, there was no association between exacerbations and lung function in any age group. In that study, treatment with low doses of ICS soon after diagnosis was found to reduce the risk of severe exacerbation and to attenuate the decline in lung function, suggesting that reducing inflammation through treatment prevents that decline.

The patients in our study began preventive treatment early after being diagnosed with asthma. The ICS was initially prescribed by pediatricians working within the Wheezy Child Program, a pioneering and successful program for the management of asthma in Brazil, which has provided training in asthma management to physicians and preventive medications free of charge since 1994.^15^ Patients in whom there was a failure of clinical control were referred to the CEMAD for further treatment. Among the patients in the CEMAD cohort, the mean time of ICS use was 6.7 years, which might have attenuated the severe exacerbations. However, because there was no comparative control group without preventive treatment, that cannot be confirmed. In the CEMAD cohort, lung function, stratified by exacerbation, remained stable, in a phase in which it should have been improving, suggesting that the patients were in an early plateau phase.

A previous study, using the initial CEMAD cohort data, extracted between 2010 and 2014,^14^ demonstrated that lung function parameters remained constant only in the patients who used high doses of ICS; that is, those with severe asthma. As of this writing, our cohort has been followed for 15 years. Because the exacerbations have continued over that time, despite the fact that they were less frequent and severe, there could have been a decline in lung function, given that the patients selected for inclusion in the cohort were those with persistent severe asthma. However, because the PFT parameters remained stable, we could not confirm any such decline. In a 3‐year prospective study of severe asthma in childhood and adolescence, patients also improved in terms of most severity indices and exacerbation frequency, although not in terms of lung function.^22^ Below‐normal lung function in childhood may predict a phenotype similar to that of early COPD.^23^

The onset of COPD can be attributed to failure to reach a normal plateau of lung function or to an accelerated decline in lung function.^23^ Susceptibility to COPD is largely determined by adverse events in childhood. To have a fundamental impact on the risk of airway disease in adulthood, interventions must begin early.^24^, ^25^

Remission of asthma is less likely in its severe forms than in its milder forms, although the decline in lung function over time is not greater in children with severe asthma than in those without asthma and those with mild asthma, suggesting that airway remodeling occurs early in childhood, resulting in suboptimal lung growth.^12^ Off‐treatment remission is one of the most important clinical targets in asthma. In one study of pediatric patients with severe asthma in Brazil,^26^ that outcome was achieved after treatment with biologics in half of the cases.

Exposure to tobacco smoke early in life has been associated with persistently low lung function trajectories.^24^, ^27^ Despite all efforts made toward asthma education at our facility, 26.6% of the patients in our cohort continued to be exposed to secondhand tobacco smoke in the home that was associated with lower lung function indices.

Our study has some limitations. First, the exacerbations were treated in emergency departments and we do not have access to information regarding their exact severity. However, in view of that limitation, we categorized as severe exacerbations only those for which we had a discharge summary showing that the patient had been treated with a systemic corticosteroid. A second limitation stems from the relatively small number of patients receiving omalizumab or mepolizumab within the study cohort. While the availability of these immunotherapies through the Brazilian public health system has gradually increased, the sample size remained insufficient to comprehensively assess the longitudinal impact of exacerbations on pulmonary function parameters in pediatric asthma patients. However, previous research by Le Thai et al.^28^ has demonstrated that omalizumab‐induced improvements in asthma symptoms in children were not accompanied by significant alterations in lung function, even with a concomitant reduction in corticosteroid dosage. The Omalizumab, although approved by the Brazilian regulatory agency (National Health Surveillance Agency) in 2017, was available to patients within the SUS until 2022 only for legal interventions. Mepolizumab's inclusion in the SUS formulary occurred in 2024. Our clinical service exclusively focuses on the management of pediatric and adolescent patients with severe asthma and relies entirely on the free provision of medications through the SUS.

To our knowledge, there have been no studies evaluating the association between exacerbations and long‐term lung function in children and adolescents with asthma who are treated with biologics. Furthermore, we did not evaluate lung function in infants and preschool children, because, despite the early onset of asthma, the patients were not referred to our facility until they were approximately 6 years of age. However, the children and adolescents evaluated in our study came from a well‐selected cohort of individuals with severe asthma who were treated at a structured reference center for severe asthma care. All of the patients had a well‐established diagnosis and were under regular monitoring by the same team, with several methods being applied in order to intervene in potentially modifiable factors, such as measuring adherence, minimizing exposure to allergens, and controlling comorbidities.^14^ Our study sample is representative of this specific population—that of pediatric patients with severe asthma—and we assessed PFT parameters at a maximum of 3 months after each exacerbation. There was no lack of access to controller medications, and all of the patients received preventive medication free of charge via the SUS.^15^ Brazil is a populous country of continental dimensions, which has the merit of having a national plan for free asthma medication. However, there is a need for multicenter studies with larger samples of patients with severe asthma and repeated measurements of lung function parameters, before and after exacerbations, that provide more detailed information on the severity of exacerbations and on baseline factors, such as a history of prematurity and low birth weight. There is also a need for multicenter prospective studies with continuous spirometry measurements in children and adolescents who are using currently available medications such as triple therapy (long‐acting muscarinic antagonist + long‐acting *β*_2_ agonist + ICS) and biologics.

In conclusion, in our sample of children and adolescents with severe asthma, exacerbations persisted despite regular use of and good adherence to treatment with ICS together with long‐acting bronchodilators and other controllers. However, exacerbations were not associated with reduced lung function in our cohort.

## AUTHOR CONTRIBUTIONS

**A. Z. P. Brandao**: Investigation; validation; writing—original draft; conceptualization; methodology; writing—review and editing; visualization. **L. M. L. B. F. Lasmar**: Conceptualization; investigation; methodology; validation; writing—original draft; visualization; writing—review and editing; project administration; formal analysis; data curation; supervision. **L. M. A. S. Pertence**: Writing—review and editing; visualization; validation; writing—original draft. **M. I. R. Vieira**: Investigation; validation; visualization; writing—review and editing; writing—original draft. **V. O. Ganem**: Writing—original draft; validation; visualization; writing—review and editing. **E. V. Mancuzo**: Writing—original draft; validation; writing—review and editing. **M. V. N. P. de Queiroz**: Conceptualization; methodology; validation; visualization; investigation; writing—original draft; writing—review and editing. **Gabriela Belizario Lasmar**: Writing—review and editing; writing—original draft; methodology; validation; visualization; investigation.

## CONFLICT OF INTEREST STATEMENT

The authors declare no conflicts of interest.

## Data Availability

The authors have nothing to report.
